# Identification of traits and functional connectivity-based neurotraits of chronic pain

**DOI:** 10.1371/journal.pbio.3000349

**Published:** 2019-08-20

**Authors:** Etienne Vachon-Presseau, Sara E. Berger, Taha B. Abdullah, James W. Griffith, Thomas J. Schnitzer, A. Vania Apkarian

**Affiliations:** 1 Department of Physiology, Northwestern University Feinberg School of Medicine, Chicago, Illinois, United States of America; 2 Healthcare and Life Sciences Department, IBM Watson Research Center, Yorktown Heights, New York, United States of America; 3 Department of Medical Social Sciences, Northwestern University Feinberg School of Medicine, Chicago, Illinois, United States of America; 4 Departments of Internal Medicine and Rheumatology, Northwestern University Feinberg School of Medicine, Chicago, Illinois, United States of America; 5 Department of Physical Medicine and Rehabilitation, Northwestern University Feinberg School of Medicine, Chicago, Illinois, United States of America; 6 Department of Anesthesia, Northwestern University Feinberg School of Medicine, Chicago, Illinois, United States of America; University of Cambridge, UNITED KINGDOM

## Abstract

Psychological and personality factors, socioeconomic status, and brain properties all contribute to chronic pain but have essentially been studied independently. Here, we administered a broad battery of questionnaires to patients with chronic back pain (CBP) and collected repeated sessions of resting-state functional magnetic resonance imaging (fMRI) brain scans. Clustering and network analyses applied on the questionnaire data revealed four orthogonal dimensions accounting for 56% of the variance and defining chronic pain traits. Two of these traits—Pain-trait and Emote-trait—were associated with back pain characteristics and could be related to distinct distributed functional networks in a cross-validation procedure, identifying neurotraits. These neurotraits showed good reliability across four fMRI sessions acquired over five weeks. Further, traits and neurotraits all related to the income, emphasizing the importance of socioeconomic status within the personality space of chronic pain. Our approach is a first step in providing metrics aimed at unifying the psychology and the neurophysiology of chronic pain applicable across diverse clinical conditions.

## Abstract 2

A neuroimaging study shows that psychological contributors of chronic pain are represented in patterns of resting state functional connectivity; clustering analysis of these patterns suggests the existence of subtypes of chronic pain patients with specific clinical characteristics.

## Introduction

Unraveling mechanisms of chronic pain remains a major scientific challenge. There is now strong and convincing evidence that specific brain properties contribute to the risk of developing chronic pain and that the transition to chronic pain involves brain adaptations that, to a large part, construct and mold the state of chronic pain [1,2]. Additionally, current theories suggest that pain characteristics, pain-related disability, and responses to treatment are all partially determined by psychological factors and/or personality properties [3], as well as parameters related to socioeconomic status (SES) [4]. Although the biopsychosocial (BPS) perspective is actively applied in the everyday clinical management of chronic pain [5], component properties that comprise the concept—namely biological processes (brain and body), psychological factors, and the impact of social factors on these components—have rarely been jointly studied. Thus, the relative influence of these factors on each other, as well as their independent contribution to the state of chronic pain, remains unknown. In its current form, the BPS model is built on fragmented evidence taken from different disciplines, and—remarkably—lacks integration between psychosocial components and underlying biology. Even though subgroupings of chronic pain types by psychological dimensions have been repeatedly formulated [6–10], a systematic approach of linking such concepts with brain biology has, to our knowledge, not been undertaken. Here, we unravel and interrelate components of BPS by combining psychological, personality, and SES factors with measures of resting state functional connectivity to begin to define a unified perspective of chronic pain.

There is a large body of literature demonstrating that psychological and personality factors are important contributors to chronic pain (e.g., the psychological components of BPS). Pain catastrophizing [11] and fear of pain [12] represent strong predictors of chronic pain, whereas pain acceptance is associated with resilience [13]. Moreover, neuroticism—one factor in the five-factor model of personality [14]—has been shown to moderate the association between catastrophic thinking and pain vigilance [15] and has been associated with substance abuse, anxiety, and major depressive disorder [16,17]. In contrast, optimism is usually associated with lower catastrophic thinking, higher pain acceptance, and better coping strategies [18]. Other psychological factors such as mindfulness or body awareness have also been linked with improvements in pain, mood disturbance, and disability [19,20], as well as with better coping strategies for managing chronic pain [21]. It is thus unsurprising that psychological factors are usually considered better predictors of chronic pain disability than the actual injury itself [22]. Despite their pivotal role in chronic pain, a comprehensive multidimensional characterization of psychological and personality factors in chronic pain remains unexplored. The first aim of the current study was therefore to determine how the psychological factors believed to impact persisting pain interacted with general personality factors to give rise to factors that define chronic pain traits, in a chronic back pain (CBP) cohort.

The neurobiological underpinnings of chronic pain psychology/personality also remain relatively unknown, as few studies have examined the association between such factors (usually focusing on pain catastrophizing) and brain functional properties. For instance, pain rumination has been associated with stronger functional connectivity between the medial prefrontal cortex and regions within the default mode network (DMN) in temporomandibular disorder [23]. In migraines, seed-based analyses show that pain catastrophizing is associated with functional coupling between several brain regions (posterior cingulate cortex with the dorsolateral prefrontal cortex and the insula with the hippocampus) [24]. In fibromyalgia, pain catastrophizing has been associated with increased dorsolateral prefrontal cortex activity in anticipation of pain [25]. However, these studies tested limited psychological parameters in association with brain properties from only a few predefined regions of interest. Here, our secondary aim was to decode traits of chronic pain using resting-state functional connectivity magnetic resonance imaging (fMRI), with the goal of determining if and how chronic pain traits are represented in patterns of brain activity, bridging the psychology of pain with its neurobiological framework, which we define as neurotraits. Thus, our primary hypothesis was that chronic pain traits can be determined from distributed neural representations.

Our third aim was to determine the influence of social components on traits and neurotraits by examining the association of SES with these dimensions. Many studies show that lower SES is associated with higher prevalence of musculoskeletal pain, neuropathic pain, ulcers, and sciatica across Europe and North America [4]. Consistently, the British Birth Cohort study revealed that lower SES during adulthood is associated with shoulder, forearm, low back, knee, and chronic widespread pain [26]. Consistent with the BPS model, SES and occupational hazards are important risk factors for the onset of back pain and pain disability, independent of injury occurrence or type [27,28]. We therefore hypothesized that SES measured with self-reported income, years of education, sex/gender, and race/ethnicity will be linked to the traits and underlying neurobiology.

Our results indicate four dimensions comprising chronic pain traits, only two of which were associated with back pain characteristics of CBP. We therefore studied brain functional connectivity networks for these two dimensions and demonstrate that they can be represented from distributed functional networks (neurotraits). These networks were reliable in repeat brain scans. Confirming our starting hypothesis, SES was associated with chronic pain traits and their related functional networks.

## Results

**Fig 1:**
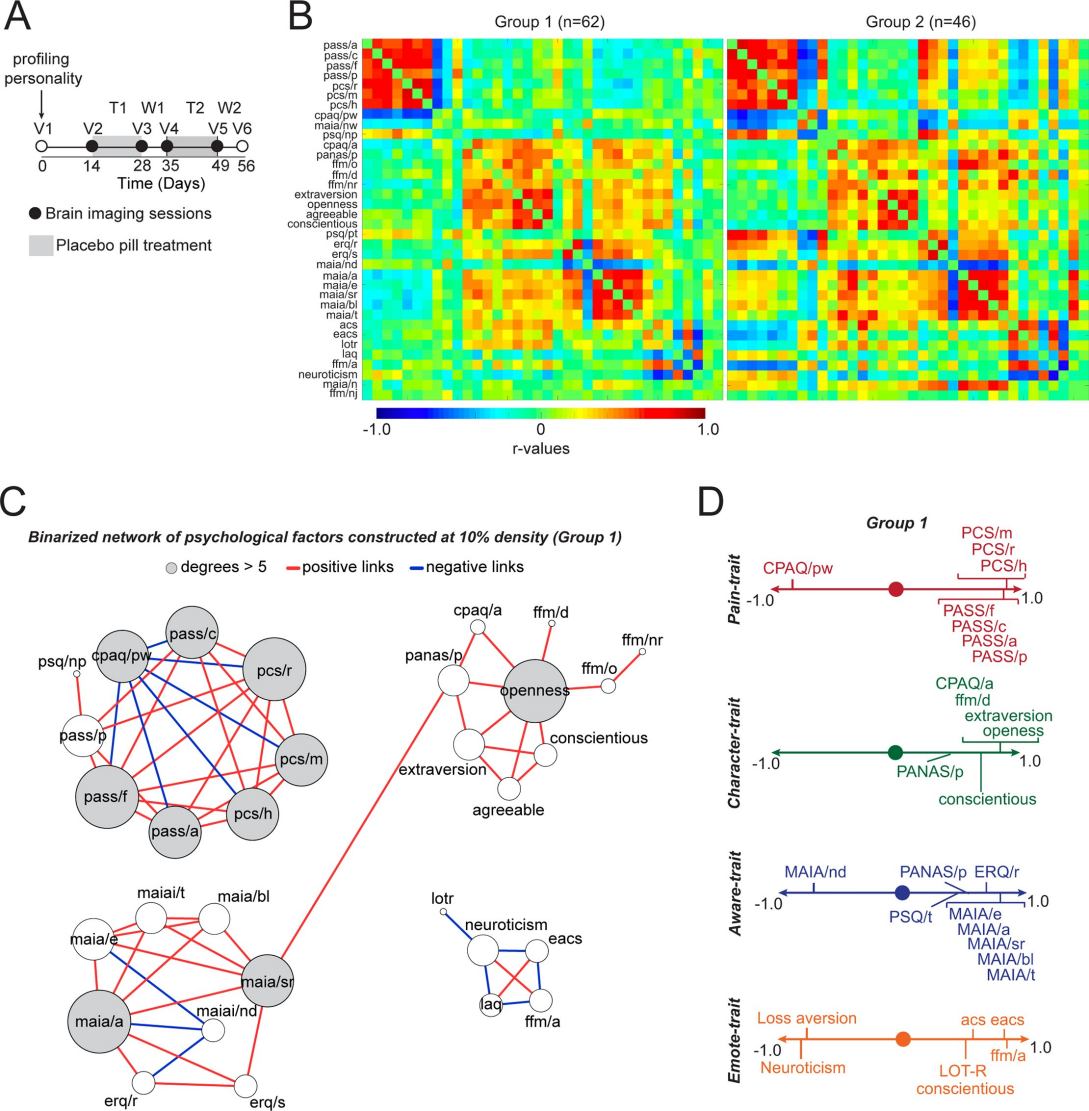
Psychological and personality factors identify four chronic pain traits. A. The study design consisted of six visits to the lab, with four of these including brain imaging sessions. Questionnaires were administered at V1 and pain was manipulated with placebo treatment (T1) followed by a washout period (W1), repeated twice (T2, W2). Sixty-two patients completed all six visits (Group 1) and 46 patients completed the questionnaires at V1 but did not finish the rest of the study (Group 2). B. Each covariance matrix (Group 1 and Group 2; ordered based on PCA results for Group 1) showed associations between the questionnaire outcomes used to probe CBP patients’ profiles (*r* values shown in bar from blue to red). C. A network constructed from the Group 1 covariance matrix displays the strongest correlations (top 10%) and topological properties indicated the presence of four communities. Size of the nodes are scaled based on degree count. D. PCA performed on all 36 questionnaire outcomes (Group 1) identified four orthogonal components. Given the observed loadings and network clustering, we label these components as: Pain-trait; Character-trait; Aware-trait; and Emote-trait. Only components loading >0.4 or <−0.4 are depicted. Abbreviations of the psychological factors: PASS and its four subscales: Avoidance (pass/a), Cognitive (pass/c), Fear (pass/f), and Physiological (pass/p); PCS and its three subscales: Rumination (pcs/r), Magnification (pcs/m), and Helplessness (pcs/h); CPAQ and its two subscores: Activity Engagement (cpaq/a) and Pain Willingness (cpaq/pw); MAIA: Not Distracting (maia/nd), Attention Regulation (maia/a), Emotional Awareness (maia/e), and Self-Regulation (maia/sr), Noticing (maia/n), Not Worrying (maia/nw), Body Listening (maia/bl), and Trusting (maia/t); PSQ and its two subscales: Painful (psq/p) and Non-Painful (psq/np); PANAS with Positive (panas/p) subscale; FFM with its five subscales: Observe (ffm/o), Describe (ffm/d), Act with Awareness (ffm/a), Non-Judge (ffm/nj), and Non-React (ffm/nr); ERQ with its Reappraisal (erq/r) and Suppression (erq/s) subscales; ACS; EACS; LOT-R; and LAQ. For each figure, the numerical data are available S1 Data, and a description of the analyses and *P* values are reported in S4 Table. ACS, Attentional Control Scale; CBP, chronic back pain; CPAQ, Chronic Pain Acceptance Questionnaire; EACS, Emotional Attentional Control Scale; ERQ, Emotional Regulation Questionnaire; FFM, Five Facets of Mindfulness; LAQ, Loss Aversion Questionnaire; LOT-R, Life Orientation Test, Revised version; MAIA, Multidimensional Assessment of Interoceptive Awareness; PANAS, Positive and Negative Affect Scale; PASS, Pain Anxiety Symptoms Scale; PCA; PCS, Pain Catastrophizing Scale; PSQ, Pain Sensitivity Questionnaire.

This study is based on data collected in the setting of a Randomized Control Trial (RCT) designed to study placebo pill response in chronic pain patients [29]. Patients with CBP enrolled in the RCT visited the lab on six occasions with four visits including brain-imaging sessions (Fig 1A). A total of 108 CBP patients were included. Group 1 included 62 CBP that completed all six visits of the RCT and were used to determine the components of personality, derive underlying patterns of brain connectivity. Group 2 included 46 CBP patients that completed all questionnaires at V1 but did not complete the brain imaging sessions for various reasons, including claustrophobia, meeting an exclusion criterion at a later point in the trial, or dropping out of the study. The demographics for these patients can be found in S1 Table. For each figure, numerical data are available in S1 Data, and a description of the analyses and *P* values are reported in S4 Table.

### Psychology/personality traits of chronic pain

We first sought to define the psychology/personality factors of chronic pain based on responses to a broad battery of questionnaires in two CBP cohorts (Group 1 and Group 2; S2 Table). We applied clustering and network analysis methods to the covariance matrix constructed from responses to questionnaires that surveyed concepts related to chronic pain; constructs of personality; and emotional, attentional, and motivational properties, all of which are thought to influence traits of chronic pain. We profiled the CBP patients using the following measures: pain anxiety (PASS), pain catastrophizing (PCS), pain sensitivity (PSQ), pain acceptance (CPAQ), emotion regulation (ERQ), attentional capacities (ACS), attentional capacities in the presence of emotions (EACS), optimism (LOT-R), sensitivity and aversion to loss (LAQ), positive affect (PANAS/p), interoceptive awareness capacities (MAIA), mindfulness capacities (FFM), and personality traits (NEO-5: neuroticism, extraversion, openness, conscientiousness, and agreeableness). The associations among subscales are presented in Fig 1B for both groups of patients. Principal component analysis (PCA) was used to order the 36 subscales in Group 1 and subsequently used to organize the questionnaires for Group 2 in Fig 1B. As shown in the figure, the pattern of covariance was very similar in the two groups.

We used Group 1 data to construct a network from the covariance matrix of 36 subscales. This network exhibited high-dimensional topology, as community clustering of the subscales segregated into four distinct communities (Fig 1C). PCA analysis applied on the covariance matrix for Group 1 also identified four orthogonal principal components with eigenvalues > 2.0 and explained more than 5% of the variance (representing the elbow in the scree plot, S1A Fig). The four components together explained 56% of the variance. PCA performed for Group 2 data identified the same four components as seen for Group 1 data, explaining 57% of the variance, validating the four dominant psychological dimensions related to chronic pain. These components are dubbed “chronic pain traits,” which we label based on the component loadings. Component 1, Pain-trait, was dominated by factors emphasizing the psychological toll of chronic pain: high positive loadings for fear of pain, pain catastrophizing, and pain anxiety and a high negative loading for pain acceptance. Component 2, Character-trait, included mainly positive personality traits such as extraversion, conscientiousness, and openness. Component 3, Aware-trait, included various awareness capacities and abilities to regulate emotions. Component 4, Emote-trait, included diminished neuroticism, lower sensitivity to loss, high optimism, strong attentional control in the presence of emotion, and high aptitude for mindfulness. Similar results were obtained in Group 2, for which the PCA identified the same four domains with factor loadings similarly as seen in Group 1 (S1B Fig). Although network and PCA analyses resulted in similar results, there were also subtle differences in the components identified by each procedure; thus, we use both representations in subsequent analyses. Overall, we identify four validated dimensions comprising traits of chronic pain in CBP patients. Unsurprisingly, the obtained pattern indicates that pain-related properties comprise the dominant dimension, while the other three dimensions comprise positive domains of character, awareness, and emotion, all of which would theoretically counterbalance or modulate the primarily negative pain-related dimension.

### Associations between chronic pain traits and back pain characteristics

We examined the interrelationship between the four trait dimensions of chronic pain with the intensity, qualities, and negative affective characteristics of the back pain experienced by our CBP patients. Our aim was to link specific traits to pain aspects of CBP. In both groups, we assessed back pain and pain-related negative affect using questionnaires and ratings. The pain intensity measurements included a series of numerical scales: out-of-clinic ecological momentary assessments (EMAs) of back pain intensity entered twice a day using smartphone technology, a verbal recall of the pain experienced over the last seven days (Memory) and an in-lab numeric rating scale (NRS) of the current pain. Pain qualities were assessed using the McGill pain questionnaire—sensory (MPQ/s) and affective (MPQ/a) subscales—and PainDetect. Negative mood and depressive symptoms were assessed using the negative affect score from the Positive and Negative Affect Scale (PANAS/n) and the Beck Depression Index (BDI-1a). Finally, physical health was also evaluated (12-item short-form survey/physical health [SF-12p]). The absolute levels in these pain measurements were equivalent between the two groups with the exception of SF-12p that was higher in Group 1. Baseline levels for these measurements are presented for both groups in S3 Table.

**Fig 2:**
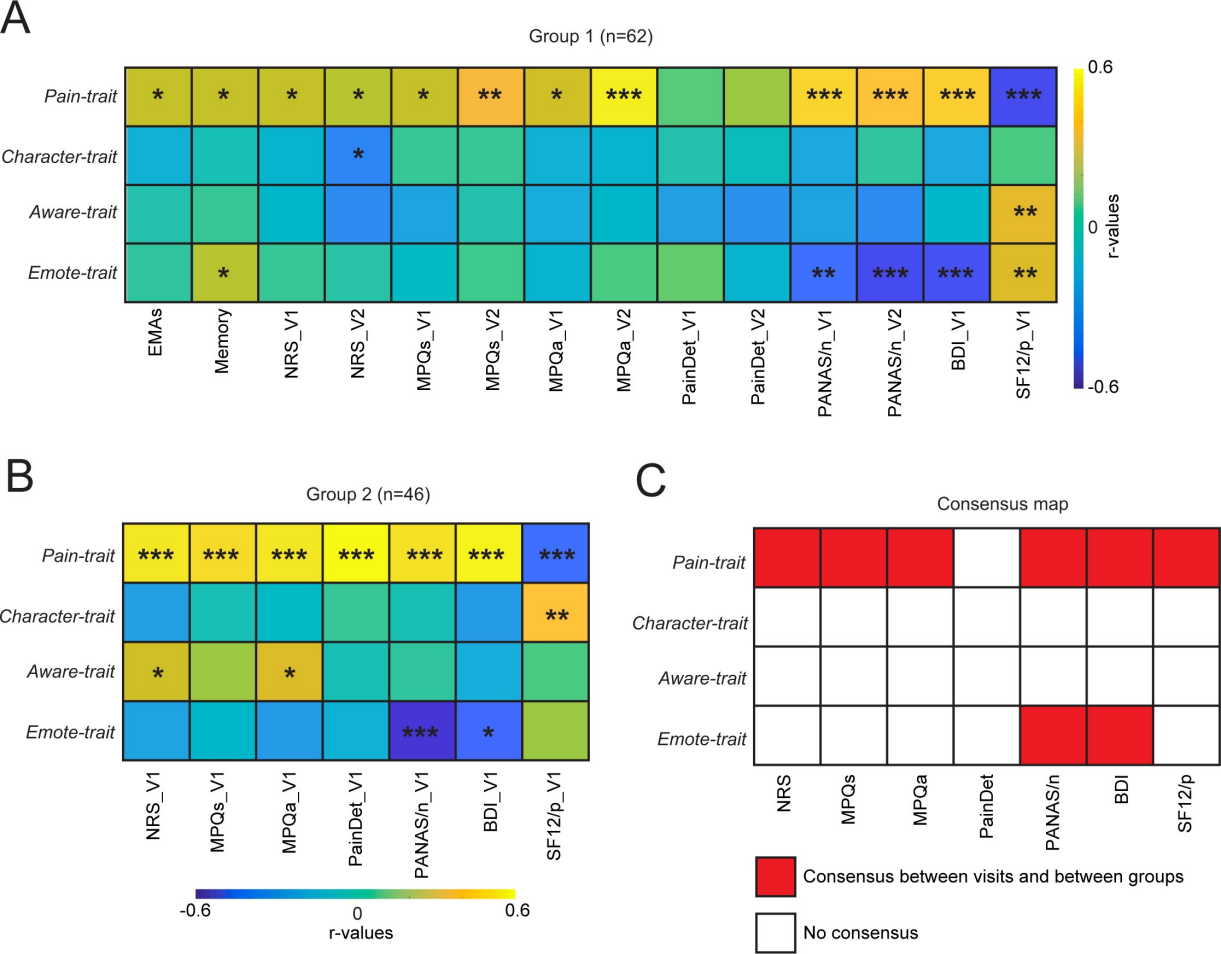
Relating trait dimensions to pain characteristics. Correlation matrices indicate the extent of the association of each trait with back pain intensity (EMAs, Memory, NRS), quality (MPQs/a, PainDetect), and negative affect (PANAS/n, BDI). A. In group 1, Pain-trait scores were related to almost all pain characteristics queried at V1 and V2, while Emote-trait scores were negatively related to negative affect at V1 and V2. B. Consistent findings were observed in group 2 (data only available at V1). C. Consensus was obtained when the effects were replicated within participant (across V1 and V2 in Group 1) and between cohort of patients (Group 1 and Group 2). The consensus map shows that Pain-trait related to almost all pain assessments and Emote-trait related specifically to negative affect. The consensus across visits and groups was established using uncorrected *P* values *P < 0.05; **P < 0.01; ***P < 0.001 (significant even after Bonferroni correction for all comparisons [56 comparisons in Group 1 and 28 comparisons in Group 2]). For each figure, the numerical data are available S1 Data, and a description of the analyses and P values are reported in S4 Table. BDI, Beck Depression Index (only collected at V1); EMA, ecological momentary assessment (phone app daily pain ratings); NRS, numerical rating scale; MPQa, McGill pain questionnaire/affective; MPQs, McGill pain questionnaire/sensory; PANASn, Positive and Negative Affect Scale/negative; SF-12p, 12-item short-form survey/physical health.

The correlation matrix between trait dimensions and pain measurements was calculated for visit 1 (V1) and visit 2 (V2) in Group 1 (Fig 2A) and for a smaller set of pain measurements for visit 1 in Group 2 (Fig 2B). Obtained results indicate that Pain-trait was positively related to almost all clinical measurements capturing back pain properties at V1 and V2, while Emote-trait was anticorrelated primarily to the negative affective characteristics of CBP (Fig 2A, Group 1). Similar associations were also observed in Group 2 (Fig 2B). Character-trait and Aware-trait showed no reliable relationships with pain or negative affect measurements. Consensus analysis between visits and groups confirmed these results (Fig 2C). Thus, we uncovered two traits associated with back pain properties, in opposite directions and with distinct patterns.

Finally, we assessed if the expression of these chronic pain traits was related with how long individuals have been suffering from persisting pain; we observed no correlation between pain duration and any traits (Pearson correlations; all *P* values > 0.24).

### Brain functional circuits defining neurotraits for Pain-trait and Emote-trait

Next, we used a connectomics-based [30,31] approach to examine if the two chronic pain traits associated with back pain properties can be decoded from functional networks using resting state fMRI in a cross-validation procedure. We used connectome-based modeling of functional connectivity to determine if brain networks underlying chronic pain traits can be identified based on the Shen et al. protocol [32]. Two different parcellation schemes were used to test the robustness of our results. First, we used the Power brain parcellation (see Methods [33]) and computed pairwise Pearson correlation coefficients between the time courses of each possible pair of 272 nodes; these generated a single-subject connectivity matrix. We then used a 3-fold cross-validation procedure, for which patients were divided into training (2/3 of patients) and testing (1/3 of the patients) sets. The training set was used to select features associated with each trait score, performing robust regressions between each link of the connectivity matrices with the specific trait score (*P* < 0.05). A leave-one-out procedure was used within the training set to reduce the number of connections to the ones correlating with the trait in each of the *n* − 1 iterations. Once the stable connections were identified, a summary statistic was calculated from the sum of all edges (*z(r)*) positively correlating with the trait and the sum of all edges (*z(r)*) negatively correlating with the trait, separately. Of note is that the edges positively correlating with the trait are not necessarily positive functional connections and vice versa for edges negatively correlating with the trait. No other approach was tested to determine brain circuitry associated with chronic pain traits.

**Fig 3:**
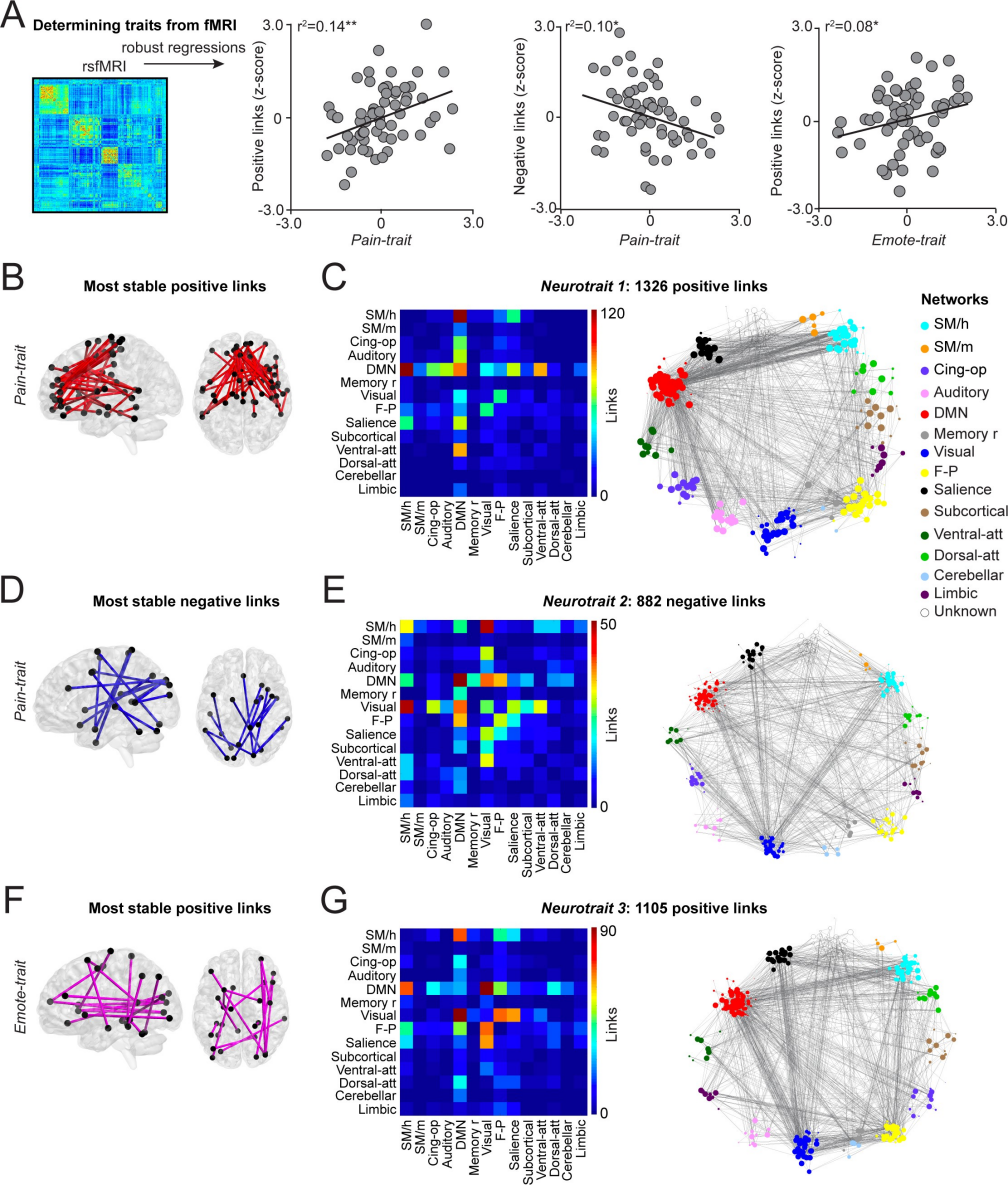
Resting state functional connectivity determined Pain-trait and Emote-trait. A. Resting-state fMRI was used to construct functional connectivity covariance matrices, and a 3-fold cross-validation procedure was implemented to identify links related to trait scores. Scatterplots show the normalized score of each individual and their chronic pain traits when they were in the left-out fold. Pain-trait was determined from both positive and negative links and Emote-trait could be determined only by positive but not negative links. B, D, F. The most stable links were the common links consistently selected by the models across all three folds. C, E, G. The matrices display within- and between-community connections of all links, irrespective of their weights, when the brain networks are represented by 14 communities. The full connectivity profile for each trait is also shown across all 272 nodes. *P < 0.05; **P < 0.01. For each figure, the numerical data are available S1 Data and a description of the analyses, and *P* values are reported in S4 Table.

The results of these correlates were validated using the left-out patients of the test set. The sum of positive and negative z*(r)* values from the same set of edges identified in the training set was calculated in the patients of the test set to determine their traits. The normalized value of each subject was correlated with their actual trait. This cross-validation procedure was repeated for the three folds, rotating and substituting the third of the patients used in the test set. A permutation test (*n* = 10,000) was used to determine significance. Fig 3A shows that three patterns of functional connectivity were successfully cross-validated using this procedure. The Pain-trait score was determined from both positive (*r*^*2*^ = 0.14; *P =* 0.002) and negative edges (*r*^*2*^ = 0.10; *P =* 0.01), and the Emote-trait score was determined only from positive edges (*r*^*2*^ = 0.08; *P =* 0.02). Because the features selected in the training set differed across the three folds, a single set of consensus weights was generated from averaging the links across the folds. Thus, an edge selected across all three folds was provided a weight of 1.0, an edge selected in two folds was provided a weight of 0.66, and an edge selected in one fold was provided a weight of 0.33. Links persistent across the three folds were labeled “most stable links” and displayed (Fig 3B, 3D and 3F).

All weighted links were organized based on their connectivity within and between the 14 communities of the human brain. The full connectivity matrices for all links related to the two traits are also shown (Fig 3C, 3E and 3G). Pain-trait scores were determined from a total of 1,326 weighted positive links: mainly between DMN and sensorimotor, cingulate, salience, and ventral attention communities (Neurotrait 1); and 882 weighted negative links: mainly between sensorimotor, DMN, memory, visual, frontoparietal, and ventral attention communities (Neurotrait 2). The Emote-trait scores were determined from a total of 1,105 weighted positive links: between DMN, visual, sensorimotor, salience, and frontoparietal communities (Neurotrait 3). Thus, highly distributed networks comprise the neurotraits related to the two chronic pain traits specifically reflecting CBP properties.

Because the traits were orthogonalized with the PCA, the edges showed little overlap between neurotraits. There was an 8% (108/1326 edges) overlap between Neurotrait 1 and Neurotrait 3 and a 6% (50/822 edges) overlap between Neurotrait 2 and Neurotrait 3. By definition, there was no overlap between Neurotrait 1 and Neurotrait 2. Further, the Emote-trait was not correlated with either Neurotrait 1 (*r* = 0.05; *P* = 0.71) or Neurotrait 2 (*r* = −0.01; *P* = 0.92) and the Pain-trait was not correlated with Neurotrait 3 (*r* = 0.09; *P* = 0.51).

The robustness of our results was then tested by interrogating a different brain atlas [34]. The same cross-validation procedure showed that brain networks constructed from the two different parcellation schemes were similar and robust for tracking Pain-trait and Emote-trait (S2 Fig). These results reveal distinct and validated neural networks corresponding to the two chronic pain-related traits.

### Neurotraits are reliable across fMRI sessions

**Fig 4:**
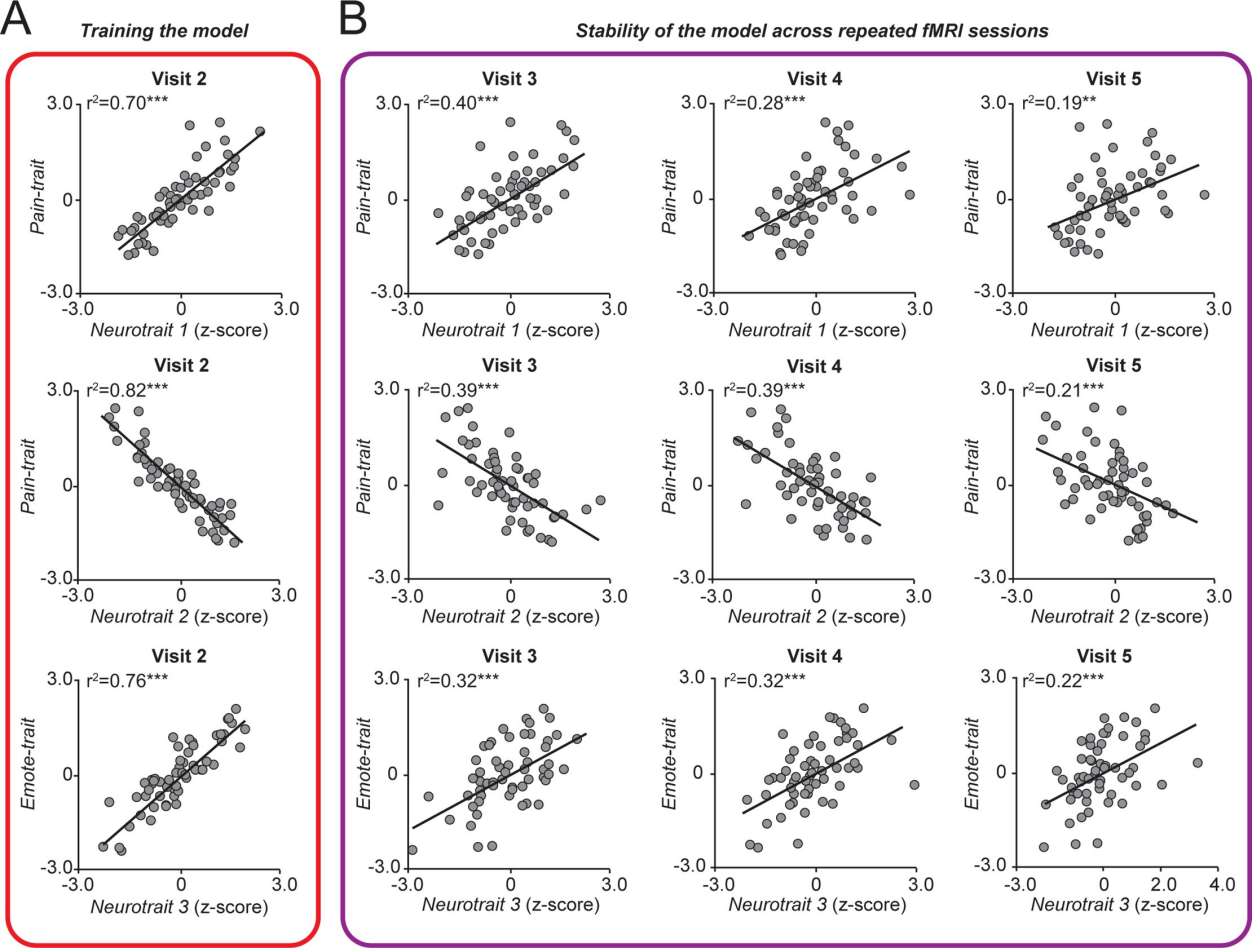
Stability assessment of neurotraits from repeat brain scans over 5 weeks. A. Strength of relationships between weighted patterns of connectivity that were trained at V2 with corresponding psychology/personality trait scores. B. Relationship between neurotraits (derived from connectivity based on subsequent brain scans) and trait scores determined by questionnaires at V1. The trait–neurotrait relationship is preserved. *P < 0.05; **P < 0.01; ***P < 0.001. For each figure, the numerical data are available S1 Data, and a description of the analyses and *P* values are reported in S4 Table.

All analyses presented thus far were performed on data collected at V1 (questionnaire data) or V2 (pretreatment brain imaging and questionnaire data). Next, we examined the stability of the three identified neurotraits by assessing functional connectivity patterns across the four longitudinal brain imaging sessions. The sum of positive and negative z*(r)* values from the same sets of edges initially identified at V2 (Fig 4A) was calculated for the resting state fMRI data collected at V3, V4, and V5 (Fig 4B). The pattern of functional connectivity calculated from V3–V5—neurotraits derived weeks after assessing the corresponding traits—successfully tracked patients’ scores on Pain-trait and Emote-trait. We further quantify the reliability of the neurotraits by computing the intraclass correlation (ICC) for each neurotrait [35]. The average ICC was Neurotrait 1: 0.87 (95% CI: Lower: 0.80; Upper: 0.92); Neurotrait 2: 0.85 (95% CI: Lower: 0.78; Upper: 0.91); Neurotrait 3: 0.83 (95% CI: Lower: 0.74; Upper: 0.89). Together, the neurotraits showed good reliability across the four brain-imaging sessions collected weeks apart.

### Associations between neurotraits and back pain characteristics

**Fig 5:**
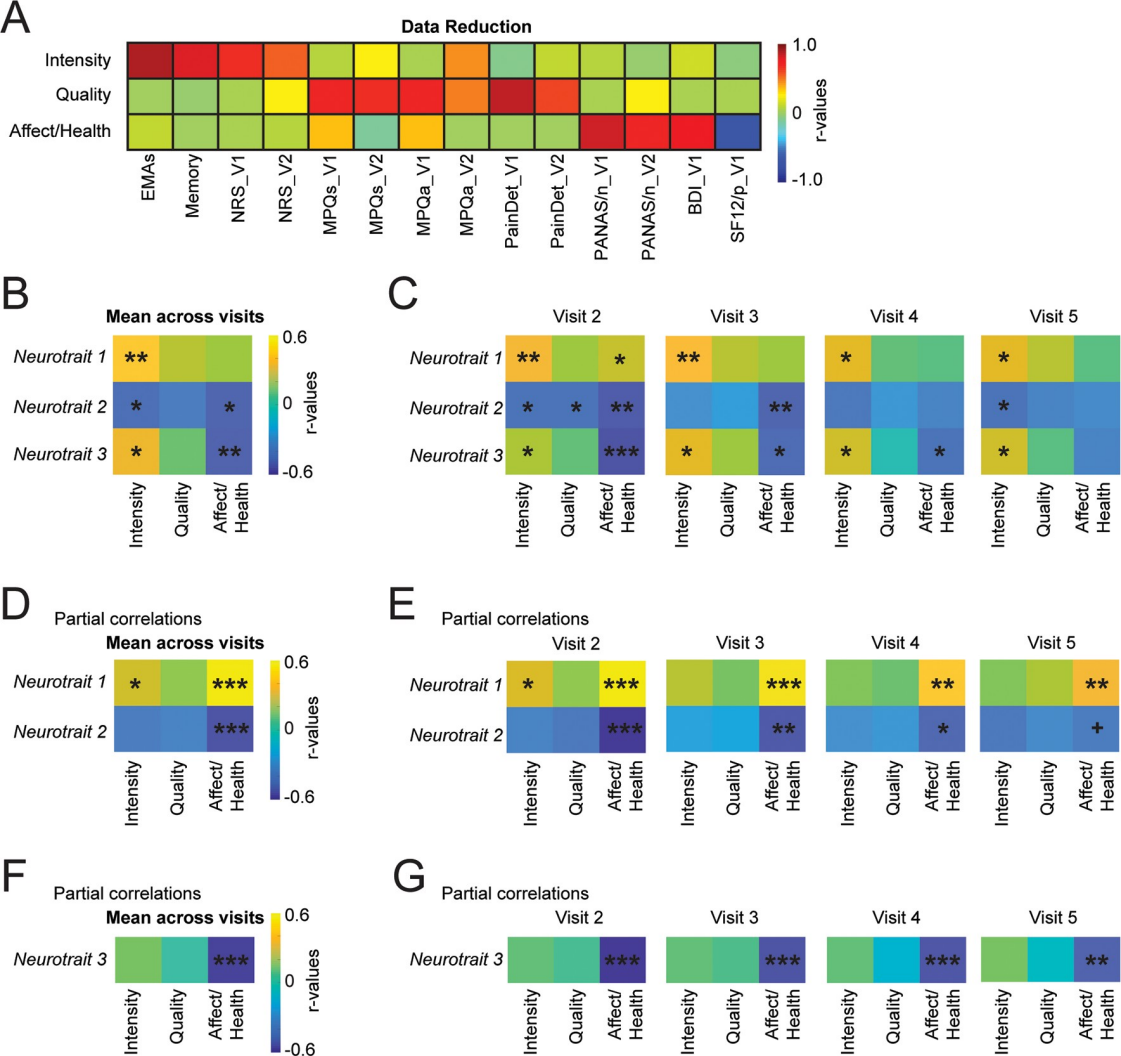
Association between neurotraits and pain measurements. A. Data reduction was performed to extract three principal components reflecting pain intensity, pain quality and affect/physical health measured at visit 1 and visit 2. The component matrix displays the correlations between the pain measurements and each component. B. The mean neurotrait represents the averaged sum of the positive and the negative z*(r)* values across all four visits (*n* = 56). The covariance matrix shows an association of the neurotraits with pain intensity and affect/health but not with pain quality. C. The covariance matrices show the associations between pain components (visit 1 and visit 2) and the neurotraits extracted at each visit separately (visits 2–5). D. Partial correlations show that Neurotrait 1 and 2 were strongly correlated with affect/negative health when controlling for Neurotrait 3. E. The effect was reliable when extracting the neurotraits from separate visits. F. Neurotrait 3 was also strongly correlated with affect/negative health when controlling for Neurotrait 1 and 2. G. The effect was reliable across each visit. ^+^*P* = 0.053; *P < 0.05; ** *P* < 0.01 (significant after Bonferroni corrections for nine comparisons); ***P < 0.001. For each figure, the numerical data are available S1 Data, and a description of the analyses and *P* values are reported in S4 Table.

We directly tested the associations between the identified brain networks (Neurotraits) and the clinical characteristics of the CBP patients (Group 1). We used data reduction on pain measurements (ratings and questionnaires, parameters shown in Fig 2A) to limit the number of analyses. We applied PCA and derived three chronic pain related components, together explaining 57% of the variance, and representing: 1) pain intensity, 2) pain qualities, and 3) negative affect and physical health (Fig 5A). Correlations and partial correlations were used to assess the associations between the neurotraits and these three chronic pain defining components.

To increase the reliability of our results, we averaged each neurotrait across the four resting-state fMRI sessions. Fig 5B shows that the three neurotraits correlated with the pain intensity and that Neurotrait 2 and Neurotrait 3 correlated with the affect/health component. However, only Neurotrait 1 with pain intensity and Neurotrait 3 with affect/health survived multiple comparisons. There were no significant associations between Neurotraits and the pain quality. Testing the association between the chronic pain components and the Neurotraits from each brain imaging session separately showed relatively consistent results, although the effect on affect/health was no longer present at visit 5 (Fig 5C).

Next, a series of partial correlations were performed to fully understand the interplay between the Neurotraits. The unique contribution of Neurotrait 1 and Neurotrait *2*, after controlling for the effect of Neurotrait 3, showed a strong association with affect/health (Fig 5D). The unique contribution of Neurotrait 3, after controlling for Neurotrait 1 and Neurotrait 2, also showed a strong association with affect/health (Fig 5F). These findings survived multiple comparisons and were consistent across visits when tested separately (Fig 5E and 5G). Our results suggest a weak nonspecific association between the three Neurotraits and the pain intensity, which mostly dissipated when applying partial correlations. On the other hand, the partial correlations revealed strong and specific associations between neurotraits and affect/health. These findings are consistent with Pain-trait and Emote-trait being independently associated with negative affect and physical health, implying that the brain networks were directly related with the clinical characteristics of the patients.

### Associations between traits, neurotraits, and socioeconomic status

**Fig 6:**
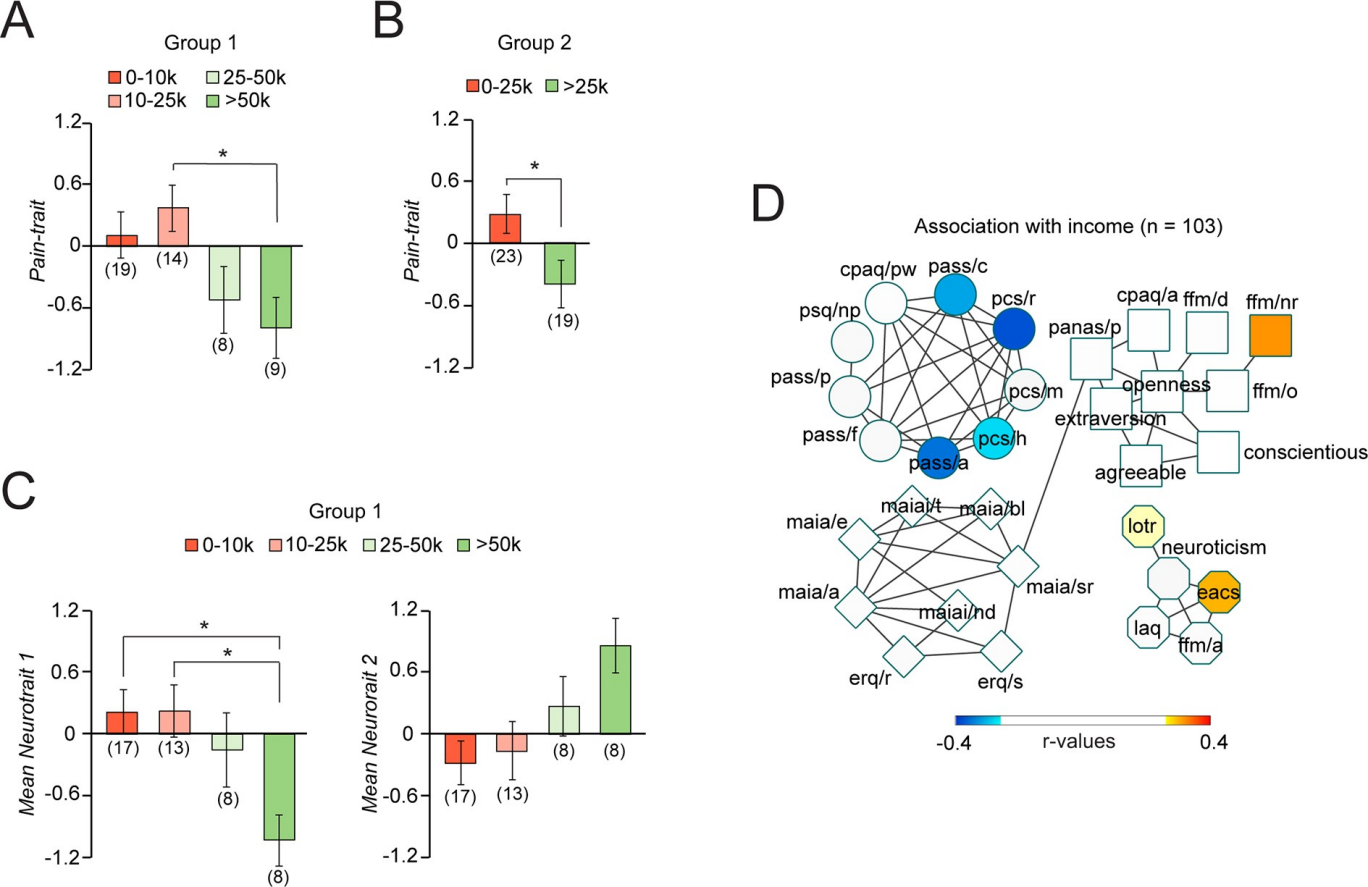
Association between socioeconomic factors and traits and neurotraits. A. Income differentiated patients on Pain-trait after controlling for ethnicity and years of education (while the inverse was not true), suggesting that socioeconomic status (rather than ethnicity or years of education) contributed to Pain-trait 1. Annual income of >25,000 seemed to represent a socioeconomic threshold distinguishing vulnerability from protection. B. Applying the 25,000 threshold yielded similar results in Group 2. C–D. Self-reported income further differentiated Neurotrait 1 and Neurotrait 2 (marginally significant; *P* = 0.06). E. Spearman correlations were used to display subscales associated with income (only significant relations are displayed (uncorrected *P* < 0.05). All abbreviations are defined in Fig 1. Post-hoc comparisons were Bonferroni corrected for six comparisons ^&^*P* ≤ 0.1; *P < 0.05. For each figure, the numerical data are available S1 Data and a description of the analyses and p-values are reported in S4 Table.

The biopsychosocial components of chronic pain have repeatedly been linked to SES. Here, we measured SES using self-reported income (US dollars earned yearly), race/ethnicity, sex/gender, and years of education separately. The years of education and the race/ethnicity (but not sex/gender) were strongly associated with income (S3 Fig) but not with the traits or neurotraits (ps > 0.13). The income was most strongly associated with chronic pain traits (when controlling for race/ethnicity and years of education), as patients reporting income greater than 25,000 expressed a lower level of Pain-trait (Fig 6A). This finding was replicated in Group 2 (Fig 6B). Moreover, the effect was not limited to psychological factors, as income was strongly linked with both positive and negative connections of Neurotrait 1 and Neurotrait 2 (Fig 6C), showing the association between SES and the individual's psychology and underlying neurophysiology. As displayed in Fig 6D, income mostly correlated with the subscales defining the Pain-trait and Emote-trait, as lower income was linked with higher anxiety and catastrophizing and higher income linked with higher optimism.

### Associations between traits and responses to treatment

We next assessed how the uncovered chronic pain traits can potentially be related with responses to treatment. In the current study, we explored how the traits related to the response to placebo pills in the settings of a RCT. We calculated the magnitude of placebo response as the strongest response (% change in pain) in either of the two treatment periods, as was previously done [29]. Interestingly, the two traits reflecting back pain properties of CBP, Pain-trait, and Emote-trait, were unrelated to the placebo response. In contrast, Character-trait and Aware-trait predisposed patients to the future magnitude of placebo response (S4 Fig). The current result highlights the fact that chronic pain traits unrelated to pain measurements are still relevant when studying other characteristics of patients, such as the likelihood of responding to a specific treatment.

## Discussion

Current work is aimed towards defining the psychological/personality traits of chronic pain and integrating these properties with brain function. We identified four chronic pain traits, which accounted for about 60% of the variance generated across a high-dimensional personality/psychology space (36 subscales from 13 questionnaires), validated in a separate sample. Only two of these traits were associated with the pain characteristics, in specific and opposing patterns. These traits were used to identify underlying brain circuitries (neurotraits), which were relatively stable over multiple weeks. Identified traits and neurotraits showed an association with socioeconomic factors—specifically, income—with the patients reporting higher income being more protected. Thus, our study provides a characterization of the chronic pain patient along psychological, neurobiological, and social dimensions, all underpinning specific traits and their derived brain networks.

Here, we advance a novel approach for deriving multivariate dimensions of chronic pain. By interrogating patients within a high-dimensional personality/psychology space—spanning the domains of attitudes toward pain (acceptance, catastrophizing, anxiety, sensitivity), interoceptive awareness, emotional regulation, and attentional control, mindfulness skills, personality (extraversion, agreeableness, conscientiousness, neuroticism, and openness), optimism, motivation (loss aversion), and positive affective state—and then applying dimensionality reduction techniques (cluster and network analyses), we uncover primary chronic pain traits and expound on their properties. Given that the dimensionality reduction methods used here depend on the input parameter space and the size of the population used, obtained results remain somewhat arbitrary and one worries about missing critical dimensions. Nevertheless, our ability to validate the effects of traits on chronic pain measurements in an independent group of patients and show that the uncovered neurotraits were relatively stable across four consecutive brain scans enhances confidence that the studied dimensions are robust, meaningful, and biologically grounded characteristics at least for neuropathic chronic back pain.

### Traits of chronic pain

The current study systematically delineates the state space of the biopsychosocial model for chronic pain. In this regard, the specific terminology that we invoke aims to highlight the limits of the biopsychosocial model as explored here. Although the biopsychosocial model has been advocated for over 30 years and is widely used in clinical pain settings (mainly in multidisciplinary pain management programs) [36–38], it still remains a poorly-defined concept [39].

Here, we begin to better understand and depict the critical biopsychosocial elements for CBP. Components of Pain-trait are all considered fundamental constituents of the biopsychosocial model. They have been repeatedly shown to be important contributors to chronic pain, and even seem to determine the analgesic efficacy of diverse therapies (topical or oral analgesics, cortisone and other injectables, surgical outcomes, and psychosocial treatments like cognitive behavioral therapy) [39]. The next two factors, Character-trait and Aware-trait, reflect positive character as well as awareness and emotional regulation traits and showed no consistent relationship with back pain properties. The fourth, Emote-trait factor, reflects higher optimism, mindfulness capacities, and lower neuroticism and loss aversion, and it was inversely related with negative affect. Thus, factors 2–4 seem to counterbalance the aversive toll of chronic pain (factor 1) and as such may be an important compensatory mechanism that patients use to tolerate the persistent CBP state (diminishing their perceived disability while living in chronic pain). From this viewpoint, Emote-trait specifically seems to offset and counteract the negative emotional impact of chronic pain. Moreover, given that factors 2–3 predetermined response to placebo treatment, and since we have shown (based on this same data [29]) that placebo response is predetermined from personality and brain anatomy and functional connectivity, we propose that the trajectories of personality reorganization with development of chronic pain, that is the interplay between factor 1 and factors 2 and 3, in turn determines CBP placebo response types.

Throughout this manuscript, we refer to the PCA factors comprising psychological and personality outcome measures as “traits.” Three of these factors (2–4) are composed of fundamental personality attributes and emotional characteristics observed in the population at large and are unlikely to represent risk factors for developing CBP, as no such evidence has been described in the past and the concept makes little mechanistic sense. The pain-related factor is, however, comprised of outcomes specific to chronic pain patients, which would by definition be changing over time, as pain transitions from an acute to a chronic condition (in this phase perhaps all four factors may be more fluid and reorganizing, paralleling brain reorganization). Further, given that chronic back pain is a negative affective condition that can last for decades or even a lifetime, associated dominant personality factors must be considered stable and thus traits but may also be undergoing fluctuations in response to new experiences painful and otherwise. We propose that the identified factors should be “cardinal” or “central” traits of chronic back pain as we expect that they dominate and shape these patients’ everyday behaviors [40].

Still, the approach raises new and critical questions that require further study. First, there is a general consensus that the human population at large is composed of five broad personality traits, also known as the Five-Factor-Model [14]. Our results show that most of these traits are clustered together in CBP (Character-trait), while other aspects (Emote-trait) become differentially related to chronic pain. Importantly, while subjective well-being is observed in individuals scoring high in extraversion and low in neuroticism in the population at large [41], in CBP, these characteristics load on orthogonal factors (that is, they vary independently) and relate to distinct properties. How the CBP traits (and their interrelationships) change and congeal in the transition from acute to chronic pain is unknown and will be exciting to explore. It will also be important to study the role of traits and individual differences in traits in the context of treatments for chronic pain. Such knowledge would be important to know from a risk-management viewpoint; concurrently, knowledge regarding malleability of these traits could provide targets for psychological or cognitive behavioral interventions. Second, we do not know the extent of invariance in the identified traits across different clinical chronic pain conditions (e.g., osteoarthritis, fibromyalgia, phantom pain, complex regional pain syndrome, pelvic pain, etc.), many of which are known to differ in their underlying biology (e.g., somatic versus visceral conditions). We also do not know how the covariance (network architecture) between these traits may change after different kinds of treatments or therapies. Further studies exploring these issues are certainly warranted.

### Neurotraits of chronic pain

Data-driven methods provide an unbiased and optimal way to map distributed brain representations for complex phenomena [42], and such an approach is especially appropriate when studying chronic pain since recent evidence indicates that pain patients exhibit a disruption of functional connectivity throughout the cortex [43]. We show that Pain-trait and Emote-trait can be determined from distributed patterns of functional connectivity in left-out-patients. The identified network (neurotraits) were validated using two independent brain atlases, showed good reliability across four brain imaging sessions, and directly related with the clinical pain measurements. Unsurprisingly, identified networks (neurotraits) involved brain regions not necessarily related to acute or chronic pain. The networks did still include a large number of connections that included the prefrontal cortex and the DMN, especially Neurotrait 1, consistent with earlier evidence showing involvement of these regions in chronic pain and catastrophizing [23,44–46].

Our results support the existence of multiple brain systems contributing and modulating the chronic pain experience. These systems involve different circuitry representing a different mixture of psychological factors and personality traits. This is in accordance with the literature indicating that several systems may contribute to chronic pain. For instance, several studies have indicated that the DMN linked with pain catastrophizing determines pain intensity [23,44–46], whereas the mesolimbic system linked with reward/punishment modulates pain perception and determines risk for transition to chronic pain [47–51]. Accordingly, we demonstrate that psychological components of Pain-trait (pain catastrophizing, anxiety, acceptance) determined vulnerability or protection (association with all pain measurements) and Emote-trait (neuroticism, sensitivity to loss, optimism, and mindfulness capacities) determined resilience (association with negative affect and physical health). Furthermore, we show that such a concept of resilience is complex and can be determined by multiple independent traits and neurotraits. Thus, these findings suggest multiple dimensions of personality interacting to determine unique chronic pain experiences that each have their own biology. Future studies should also incorporate neuroanatomical and structural brain properties, as these factors may further enhance the explanatory power of the neurotraits in relation to corresponding psychological/personality traits of CBP.

### Socioeconomic influences on traits and types of chronic pain

Chronic pain traits and neurotraits were specifically associated with income. One may conclude that these are at least partially interlinked with environmental factors beyond the SES variables studied here to also include upbringing, culture, and politics. Our results replicate previous research findings showing that lower incomes related with higher pain scores but also increased disability resulting from chronic pain [52]. Importantly, SES was most strongly linked with subscales defining the traits of patients, as income correlated with components of Pain-trait (catastrophizing and anxiety of pain) and Emote-trait 4 (optimism). Moreover, the association between income with Neurotrait 1, and Neurotrait 2 further enhances confidence in the consistency and interpretability of the identified brain networks reflecting the traits. Thus, the link between income and our traits provides insights into the social component of the biopsychosocial model and further substantiates the idea that chronic pain experience is not only rooted in biology but also intimately embedded in society.

In an earlier preprint version of this manuscript (https://www.biorxiv.org/content/10.1101/421438v1), we reported on deriving subtypes (which we defined as neuropsychotypes) of CBP based on clustering the CBP patients using neurotrait measurements. Further analyses (as suggested by a reviewer) indicated that the subtypes were not stable, pointing that such categorizations were arbitrary. Therefore, these results were removed in this version of the manuscript.

### Pitfalls and limitations

The current study included cross-validation of the neurotraits, assessment of within-subject robustness of neurotraits, and a validation cohort without brain imaging. Despite these strengths, the total number of subjects remains relatively small (*n* = 108: *n* = 62 with brain imaging, and *n* = 46 without brain imaging). Further, patients were excluded if they showed moderate or severe depressive symptom severity, which may influence the generalizability of the results. Moreover, the biological variables were restricted to resting state functional connectivity derived from brain imaging, whereas variables from the periphery or central spinal processing were not included. Finally, the social variables were restricted to sex/gender, race/ethnicity, years of education, and income, while other variables such as working environment, city living, marital status, and access to healthcare were not included or collected, even though they likely also play a role in the expression of pain traits or neurotraits. Therefore, future studies should include additional variables to fully examine the interplay between the neurobiological, psychological, and social components of chronic pain.

### Conclusions

We advance a methodology for identifying traits in neuropathic CBP, but the dimensions used to develop the trait space are globally relevant to other chronic pain conditions. Besides providing a comprehensive specification of the biopsychosocial properties of CBP, the approach provides tools and metrics that enable direct comparisons between such conditions regarding the linkage between neurophysiology and the psychology of chronic pain. Future studies are, however, necessary to replicate the identified traits and neurotraits, test generalizability across chronic pain conditions, and determine their clinical utility.

## Material and methods

### Ethics statement

The research has been approved by the Northwestern University IRB (ID: R1018-STU80585) in accordance with the principles expressed in the Declaration of Helsinki. Written informed consent was obtained for each participant.

### Study design

This study was conducted in the setting of a clinical RCT specifically designed for assessing the placebo response (ClinicalTrials.gov: NCT02013427). The results of the placebo response have been previously published [29], and this is a reanalysis of the same data set. The study consisted of six visits spread over approximately 8 weeks, including a baseline monitoring/screening period and two placebo treatment periods, each followed by a washout period. A full description of the study procedure on each visit, as well as the results of the placebo manipulation, can be found in [29]. Here, we restricted our initial analyses to questionnaire and brain-imaging data collected prior to placebo treatment. However, the brain imaging data acquired after the placebo treatment was used to test the stability of cross-validated patterns of functional connectivity.

### Study population

One hundred twenty-nine participants with CBP were initially recruited from the general population via advertising in the community and clinical referrals via hospital databases. Patients were assessed for general eligibility via self-report using a screening intake form that the lab. Individuals had to be 18 years or older with a history of lower back pain for at least 6 months. This pain should have been neuropathic (radiculopathy confirmed by physical examination was required), with no evidence of additional comorbid chronic pain, neurological, or psychiatric conditions. The pain level had to be of at least 5/10 during the screening interview, and the average pain level from the smartphone app needed to be higher than 4/10 during the baseline rating period. Patients with BDI score > 19 were excluded from the RCT. Finally, clinical measurements taken at V1 were required to be within the pre-specified healthy range [29] and all participants passed the MRI safety screening requirements at each scanning visit.

Of the enrolled 125 patients, 63 chronic pain patients completed the study. One of these patients was removed from the brain imaging analyses because it failed quality control (see below). Forty-seven additional patients completed all questionnaires from V1, but did not complete the brain imaging session because they failed to meet the inclusion criteria at V1 (BDI score > 19; *n* = 4), showed average pain levels lower than 4/10 during the 2-week baseline period between V1 and V2 (*n* = 16), were claustrophobic or MRI incompatible (*n* = 9), had out-of-range vital signs or blood lab results (*n* = 6), or discontinued the study (e.g., were lost to follow-up, had increased pain, or no longer wanted to be in the study) (*n* = 11). Participants were compensated US$50 for each visit completed, and they were reimbursed up to US$20 for travel and parking expenses if applicable.

### Software used for data analyses

Statistical analyses including data reduction (PCA), test for normality of the data (using Shapiro–Wilk test), group comparisons when the data was normally distributed (t test and ANCOVA), Pearson and Spearman correlations, and Intra class correlations were all performed using SPSS version 21.0.0. Preprocessing of the brain imaging data was performed using in house Matlab codes (version 2017b) and FSL version 5.0. Connectome based modeling was performed using Matlab. Modularity analysis applied on the questionnaire data was performed with the Network analyser app in Cytoskape (https://cytoscape.org/) version 3.6.1.

### Ecological momentary assessments using phone app

Each patient’s pain was monitored electronically using an app designed specifically for the study. The app had a VAS scale with sliding bars on which the participant was asked to rate their current pain level from 0 (no pain) to 10 (worst imaginable). Participants were instructed to use the app twice a day and were compensated $0.25 for each rating they submitted, up to $0.50/day. This additional payment was given at the end of the study to encourage compliance throughout the study. The ratings were sent to a secure server and both date- and time-stamped. The overall compliance of the phone ratings was on average 76%.

### Other chronic pain measurements

We assessed chronic pain intensity using multiple measurements. Besides the EMAs, a numerical rating scale and a verbal recall of the averaged pain experienced over the last week (memory) was collected in the lab. Second, the qualities of the pain were assessed using the MPQ affective and sensory subscales, and PainDetect. Third, the mood was assessed with PANAS/negative subscale and the Beck Depression Inventory, Version 1a (BDI-1a). Finally, we assessed physical health using the SF-12p physical subscale.

### Analysis of questionnaire data

A full list of all 13 questionnaires used to measure psychological and personality factors can be found in S2 Table. Data from these self-report measures was downloaded directly from REDCap as a CSV file and scored in Excel according to their references [29]. An option to “skip” a question was provided for each item. If more than 20% of the data from a given questionnaire (or questionnaire subscale, if applicable) was missing, the person’s data for the questionnaire was not scored. For any other missing data, the missing item was replaced by the mean (if the questionnaire had sub-scoring, the mean was calculated from the remaining items in the sub-dimension as opposed to the entire questionnaire).

Data reduction was performed using two strategies. Firstly, a binarized undirected network was constructed using the strongest 10% correlation coefficients of the covariance matrix between the 36 subscales (i.e., network constructed at 0.1 density). Modules were identified using the Network analyser app in Cytoskape. Secondly, a principal component analysis was applied on the 36 subscales to extract four components explaining the above 56% of the variance. The components were orthogonalized using varimax rotation in SPSS.

### Socioeconomic status

Self-reported income was measured with the following categories: 0–10,000; > 10 < 25,000; > 25,000 < 50,000; > 50,000. Self-reported years of education was reported with a single number that ranged from 8–20. Race/ethnicity was also self-reported by the participant. There were a few missing data (patient skipped the question or preferred not to answer) for self-reported income, years of education, and ethnicity. In those cases, no attempt was made to fill those missing items. Because these missing values sometimes lead to analyses with different number of patients, the *n* are provided on each figure for each analysis.

### Brain-imaging protocol and data analysis

Brain-imaging data are the same as in [29]. The data were acquired with a Siemens Magnetom Prisma 3 Tesla. The entire procedure was completed in about 35 min, but an extra 25 min was planned to install the patients in a comfortable position and to reacquire images, if necessary.

High-resolution T1-weighted brain images were collected using integrated parallel imaging techniques (PAT; GRAPPA) representing receiver coil-based data acceleration methods. The acquisition parameters were: isometric voxel size = 1 X 1 X 1 mm, TR = 2,300 ms, TE = 2.40 ms, flip angle = 9°, acceleration factor of 2, base resolution 256, slices = 176, and field of view (FoV) = 256 mm. The encoding directions were from anterior to posterior, and the time of acquisition was 5 min and 21 sec.

Blood oxygen level-dependent (BOLD) contrast-sensitive T2*-weighted multiband accelerated echo-planar-images were acquired for resting-state fMRI scans. Multiband slice acceleration imaging acquires multiple slices simultaneously, which permits denser temporal sampling of fluctuations and improves the detection sensitivity to signal fluctuation. The acquisition parameters were: TR = 555 ms, TE = 22.00 ms, flip angle = 47°, base resolution = 104, 64 slices with a multiband acceleration factor of 8 (8 X 8 simultaneously acquired slices) with interleaved ordering. High spatial resolution was obtained using isomorphic voxels of 2 X 2 X 2 mm, and signal-to-noise ratio was optimized by setting the field of view (FoV) to 208 mm. Phase encoding direction was from posterior to anterior. The time of acquisition lasted 10 min and 24 sec, during which 1,110 volumes were collected. The patients were asked to keep their eyes open and to remain as still as possible during acquisition. The procedure was repeated two times (20.4 minutes).

#### Preprocessing of functional images

The pre-processing was performed using FMRIB Software Library (FSL) and in-house software as previously describe [29]. The first 120 volumes of each functional dataset were removed in order to allow for magnetic field stabilization. The decision to remove this number of volumes was taken arbitrarily (it was not motivated upon examination of data) and we explored no other option. This left a total of 990 volumes for functional connectivity analyses. The effect of intermediate to large motion was initially removed using fsl_motion_outliers. Time series of BOLD signal were filtered with a Butterworth band-pass filter (0.008 Hz < f < 0.1 Hz) and a nonlinear spatial filter (using SUSAN tool from FSL; FWHM = 5 mm). The six parameters obtained by rigid body correction of head motion, global signal averaged over all voxels of the brain, white matter signal averaged over all voxels of eroded white matter region, and ventricular signal averaged over all voxels of eroded ventricle region were then regressed out from the BOLD signal. These nine vectors were filtered with the Butterworth band-pass filter prior to being regressed from the time series. Multivariate Exploratory Linear Optimized Decomposition into Independent Components (MELODIC tool in FSL) was used to identified components in the time series that were most likely not representing neuronal activity. Components representing motion artifact were identified if the ratio between activated edge (one voxel) and all activated regions on a spatial component was >0.45, or if the ratio between activated white matter and ventricle and whole-brain white matter and ventricles was >0.35. Moreover, noisy components were identified if the ratio between high frequency (0.05–0.1) and low frequency (0.008–0.05) was > 1. This ICA regression procedure was kept very conservative to identify only obvious components related to motion or noise.

The functional image registration was optimized according to a two-step procedure. All volumes of the functional images were averaged within each patient to generate a contrast image representative of the 990 volumes. A common template specific to our CBP patients was generated by linearly registering these mean images to the MNI template and averaging these contrast images across all participants. All preprocessed functional images were nonlinearly registered to this common template using FNIRT tool from FSL. The registered brains were visually inspected to ensure optimal registration.

On average, relative head motion was relatively low (mean frame displacement (FD) = 0.11; STD 0.07 for the first fMRI run, and mean FD = 0.11; STD 0.09 for the second run). Importantly, between-subject head motion was not related with any of the neurotraits (see below).

#### Parcellation schemes

The main parcellation scheme was the same as the one used in [29]. The brain was divided into 264 spherical ROIs (5-mm radius) located at coordinates showing reliable activity across a set of tasks and from the center of gravity of cortical patches constructed from resting state functional connectivity[33]. In addition, 5-mm radius ROIs were manually added in the bilateral amygdala, anterior hippocampus, posterior hippocampus, and NAc. Linear Pearson correlations were performed on time courses extracted and averaged within each brain parcel. Given a collection of 272 parcels, time courses were extracted to calculate a 272 x 272 correlation matrix. These matrices were used for connectome-based modeling, as described below.

A second parcellation scheme was used to test the robustness of the cross-validation results. We used 400 ROIs from [34] varying from the Power parcellation scheme in the shape and the number of ROIs (S2 Fig). In this parcellation scheme, every voxel was included in one ROI and the ROIs were defined from task and resting state functional connectivity. Unlike the Power atlas, the Schaefer atlas did not cover the subcortical structures and the cerebellum.

#### Exclusion of participants

One patient was excluded from all fMRI analyses because of aberrant values in the correlation matrix (values were above 20 STD from the mean). This subject was a priori rejected during initial quality check and was never included in any of the analyses (including the behavioral analyses). This subject was already identified as problematic and excluded in [29].

A second participant was excluded because of signal dropout in ROIs near the ventricle. This participant was included in our previous placebo study because we previously limited our analyses to 122 a priori selected ROIs [29]. Here, we used the whole 272 ROIs and had to exclude this participant. The final sample size for brain imaging collected at V2 was therefore *n* = 61.

The longitudinal data included a total of *n* = 56 patients that passed quality check for each one of the four brain-imaging sessions.

### Connectome-based modeling

We used a validated data-driven protocol to determine behaviors (chronic pain trait scores) from brain connectivity measures between the ROIs of our parcellation scheme (i.e., the connectome). We first divided the patients into a training set (2/3 of the sample) and a testing set (1/3 of the sample). The training set was used to build the model, which was then applied to the testing set. We defined the traits using a principal component analysis applied to the full sample (as in Fig 1) and did not applied data reduction within the training set prior to applying it to the test set. The robustness of the components was instead evaluated using a totally independent group of 46 chronic pain patients without brain imaging. The consistency of the principal components across data sets can be appreciated in S1 Fig.

To train our model, we used robust regression between each one of the 36,856 connections with score on Pain-trait and Emote-trait (*P* < 0.05). We tested the stability of each using a leave-one-out procedure and selected only the edges correlating with the trait in each of the *n* − 1 iterations. The end result was a number of stable edges positively or negatively correlating with Pain-trait score and Emote-trait. These edges’ strengths were summed to generate a single value, tracking each subject’s trait score.

To determine chronic pain traits in new subjects, we extracted the summed strength of edges identified in the training set from the brain connectivity data in the testing set. This cross-validation procedure was repeated for the three folds, rotating, and substituting the 1/3 of the patients used in the test set.

Two steps were used to ensure that motion did not influence the predictive patterns of brain connectivity. The first one was performed at the edge level, where removing the edges correlating with motion did not change the results. For instance, removing the edges related to motion at an arbitrary cutoff of *P* < 0.05 yielded very similar prediction accuracy in the test set (*neurotrait 1*: *r*^*2*^ = 0.13, *P* = 0.004; *r*^*2*^ = 0.08, *P* = 0.03; *r*^*2*^ = 0.07*; P =* 0.04). Secondly, we tested the effect of motion at the individual level by regressing out interindividual head motions. Thus, the pattern of functional connectivity (neurotraits) was uncorrelated with the mean head motion (Frame Displacement, FD) (all *P* values > 0.13) and regressing out the effect of the mean FD did not change the relationship between the Neurotrait 1 with Pain-trait (without covariates *r* = 0.83, *P* < 0.001; covarying for mean FD: *r* = 0.83, *P* < 0.001), Neurotrait 2 Pain-trait (without covariates *r* = 0.91; *P* < 0.001; covarying for mean FD: *r* = 0.91, *P* < 0.001), and Neurotrait 3 with Emote-trait (without covariates *r* = 0.87, *P* < 0.001; covarying for mean FD: *r* = 0.87, *P* < 0.001).

### ICC

We computed the ICC for each neurotrait using a two-way random model in SPSS to assess the absolute consistency of the metric across all four scans.

The numerical data are available in S1 Data, the raw data are available at http://www.openpain.org/placebo_1/, and the code is available at https://github.com/EtienneVP.

## Supporting information

S1 Fig Number of components from the PCA and correspondence between the groups. A. PCA was performed in Group 1 and Group 2 separately. The components with eigenvalues greater than 2.0 (symbols in red) were retained and correlated with pain outcomes. Other components with eigenvalues below 2.0 (white symbols) were used as components of personality and no exploratory analyses were performed with these. B. The PCA performed on the same variables in Group 2 CBP identified the same four components (validation). Scatterplots show strong correspondences between loading values of each component, across the two groups (validation). CBP, chronic back pain; PCA, principal component analysis.
(TIF)

S2 Fig Cross-parcellation reproducibility. A–B. The Power atlas used to derive the neurotraits as presented in Fig 3. C. The Schaefer atlas was used as a second atlas to test the robustness of our results. D. Pain-trait was determined from both positive (*r*^*2*^ = 0.08; *P* = 0.006) and negative (*r*^*2*^ = 0.04; *P* = 0.04) links and Emote-trait could be determined by positive (*r*^*2*^ = 0.06; *P* = 0.03) but not negative links. E. The interindividual correlations between the cross-validated links from the two atlases showed that the cross-parcellation yielded robust results. This suggests that the neurotraits were not specific to our choice of parcellation scheme and provide evidence that they represent a robust metric. Significance was determined with a permutation test (10,000 permutation). *P < 0.05, **P <0.01, ***P < 0.001.
(TIF)

S3 Fig Other measures of socioeconomic status related with income (displayed when pooling Group 1 and Group 2). Income depended on race/ethnicity (A) and education (B). HA, AA, WA, HS (≤12 [yoe]), AD (12 yoe > AD < 16 yoe), BD (≥16 yoe). All post-hoc comparisons are Bonferroni corrected for three comparisons. ^&^*P* < 0.08, **P < 0.01. AA, African American; AD, Associate degree; BD, Bachelor’s degree; HA, Hispanic American, HS, high school; WA, White American; yoe, years of education.
(TIF)

S4 Fig Traits predisposing patients’ responses to placebo pills. Character-trait and Aware-trait predisposed patients to the placebo response (% analgesia). Correlation *P* values were Bonferroni corrected for four comparisons *P < 0.05.
(TIF)

S1 Table Basic demographics for both groups. Group 1 was on average 5 years younger than Group 2 (unpaired *t* test_(106)_; *t* = 4.67, *P* = 0.03). There were no significant differences between the groups for gender, pain duration, or education level.
(PDF)

S2 Table List of questionnaires administered to patients for profiling psychological factors and personality traits. Thirteen self-report measures (36 measures total if divided into respective subscales) were completed at designated visits in the study. The names and abbreviation are provided for each questionnaire, along with the rationale for why each measure was included in our battery.
(DOCX)

S3 Table Baseline values of back pain characteristics in both CBP groups prior to treatment. There were no differences in absolute scores of pain measurements between the groups, with the exception of physical health (SF-12p), which was higher in Group 1 than in Group 2. CBP, chronic back pain.
(PDF)

S4 Table Details of statistical tests for results shown in Figs 1–6. (PDF)

S1 Data Data underlying figures. (XLSX)

## Abbreviations

ACS: Attentional Control Scale
BDI: Beck Depression Index
BPS: biopsychosocial
CBP: chronic back pain
CPAQ: Chronic Pain Acceptance Questionnaire
DMN: default mode network
EACS: Emotional Attentional Control Scale
EMA: ecological momentary assessment
FFM: Five Facets of Mindfulness
fMRI: functional magnetic resonance imaging
LAQ: Loss Aversion Questionnaire
LOT-R: Life Orientation Test, Revised version
MAIA: Multidimensional Assessment of Interoceptive Awareness
MPQ: McGill pain questionnaire
NRS: numeric rating scale
PANAS: Positive and Negative Affect Scale
PASS: Pain Anxiety Symptoms Scale
PCA: principal component analysis
PCS: Pain Catastrophizing Scale
PSQ: Pain Sensitivity Questionnaire
RCT: Randomized Controlled Trial
SES: socioeconomic status
SF-12p: 12-item short-form survey/physical health
