# Supplementary figures and images for: Identification of traits and functional connectivity-based neurotraits of chronic pain
Source: PLoS Biol. 2019 Aug 20;17(8):e3000349. doi: 10.1371/journal.pbio.3000349 (PMC6701751; doi:10.1371/journal.pbio.3000349)

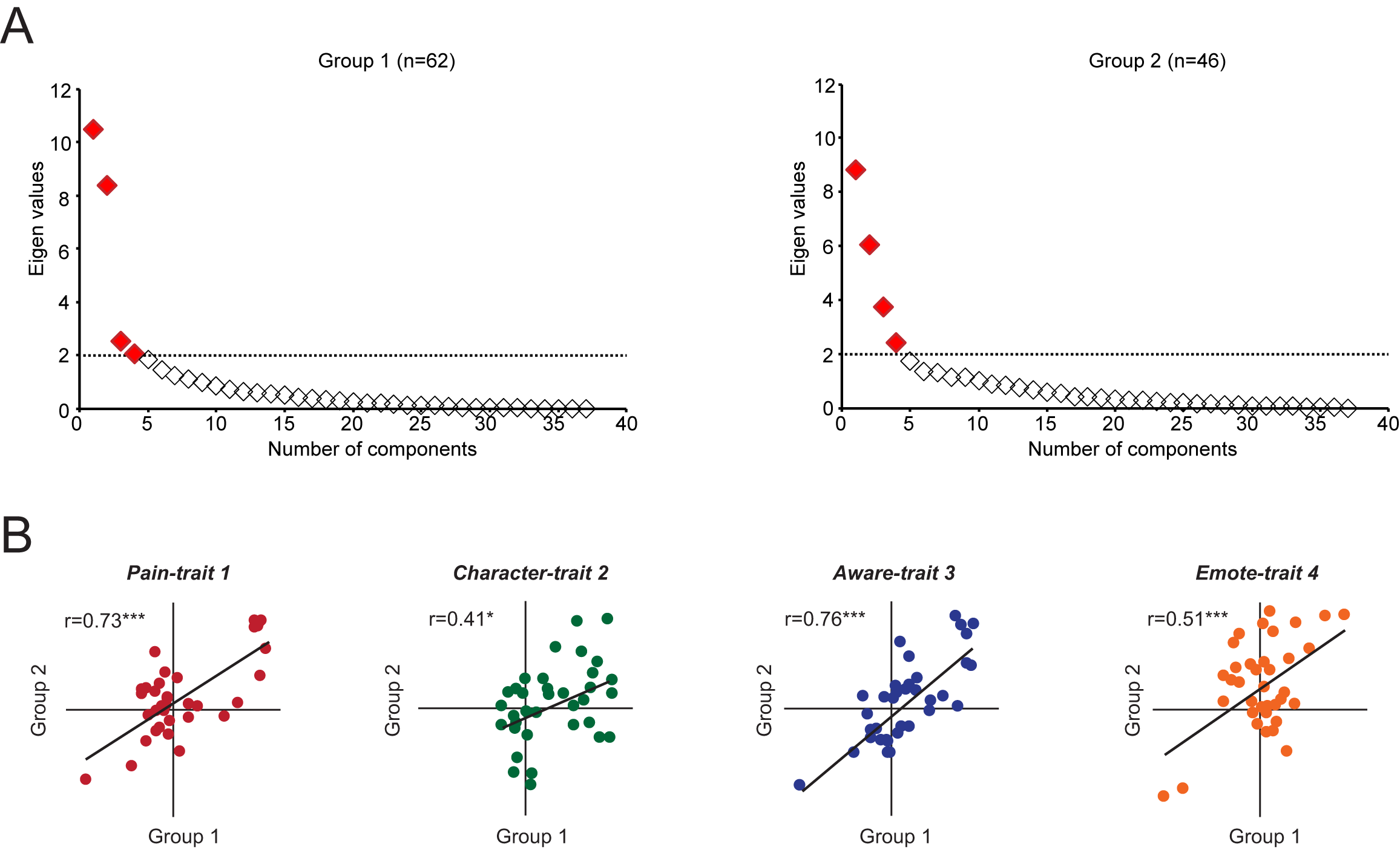

Supplement: S1 Fig — A. PCA was performed in Group 1 and Group 2 separately. The components with eigenvalues greater than 2.0 (symbols in red) were retained and correlated with pain outcomes. Other components with eigenvalues below 2.0 (white symbols) were used as components of personality and no exploratory analyses were performed with these. B. The PCA performed on the same variables in Group 2 CBP identified the same four components (validation). Scatterplots show strong correspondences between loading values of each component, across the two groups (validation). CBP, chronic back pain; PCA, principal component analysis. (TIF) [file pbio.3000349.s001.tif]

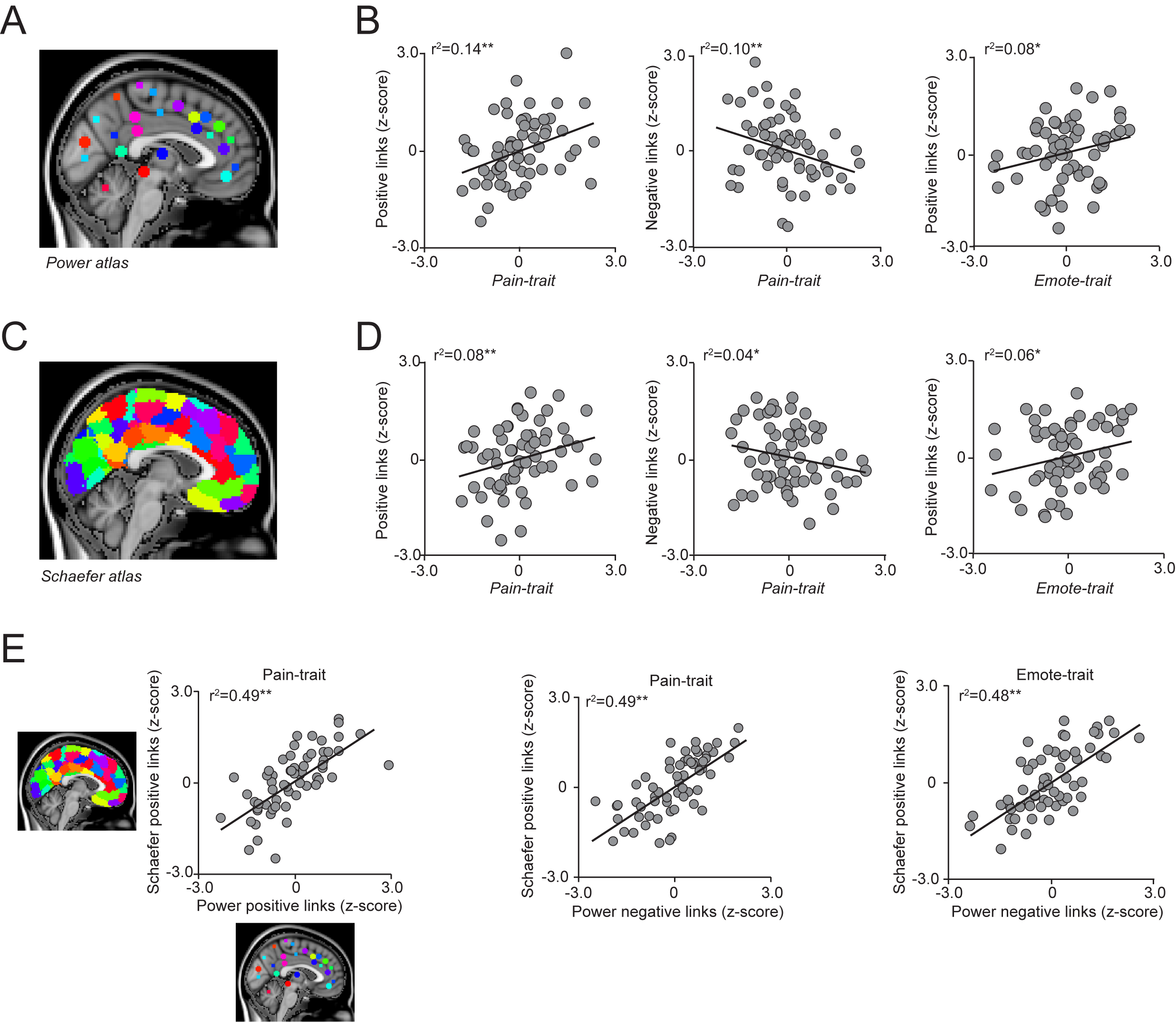

Supplement: S2 Fig — A–B. The Power atlas used to derive the neurotraits as presented in Fig 3. C. The Schaefer atlas was used as a second atlas to test the robustness of our results. D. Pain-trait was determined from both positive (r2 = 0.08; P = 0.006) and negative (r2 = 0.04; P = 0.04) links and Emote-trait could be determined by positive (r2 = 0.06; P = 0.03) but not negative links. E. The interindividual correlations between the cross-validated links from the two atlases showed that the cross-parcellation yielded robust results. This suggests that the neurotraits were not specific to our choice of parcellation scheme and provide evidence that they represent a robust metric. Significance was determined with a permutation test (10,000 permutation). *P < 0.05, **P <0.01, ***P < 0.001. (TIF) [file pbio.3000349.s002.tif]

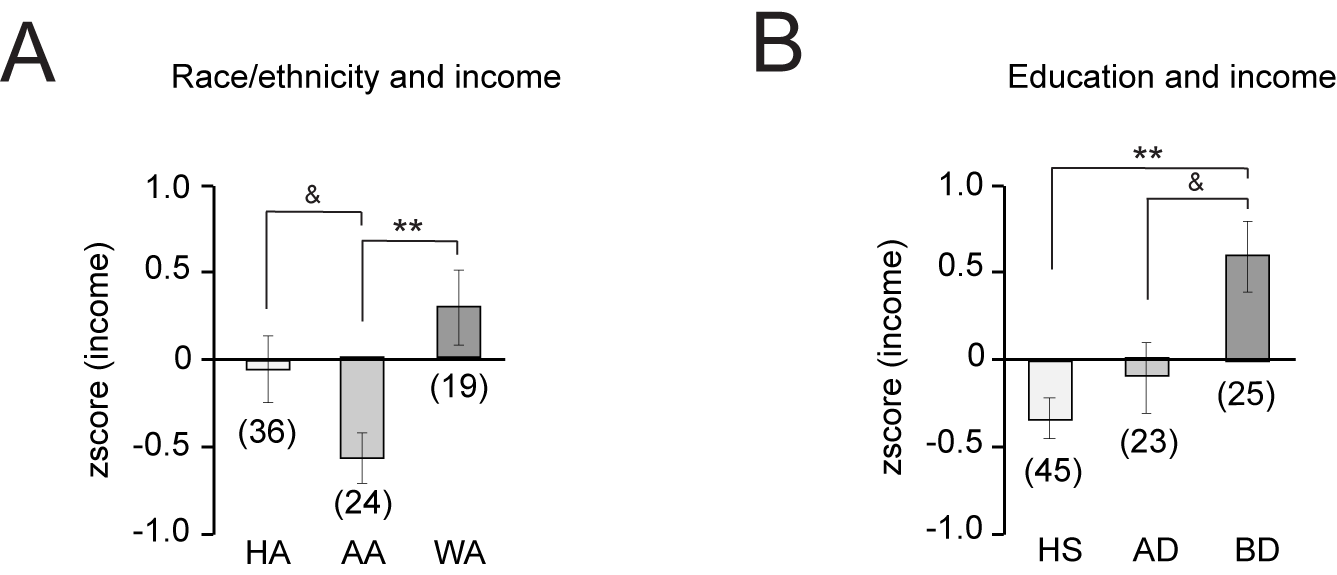

Supplement: S3 Fig — Income depended on race/ethnicity (A) and education (B). HA, AA, WA, HS (≤12 [yoe]), AD (12 yoe > AD < 16 yoe), BD (≥16 yoe). All post-hoc comparisons are Bonferroni corrected for three comparisons. &P < 0.08, **P < 0.01. AA, African American; AD, Associate degree; BD, Bachelor’s degree; HA, Hispanic American, HS, high school; WA, White American; yoe, years of education. (TIF) [file pbio.3000349.s003.tif]

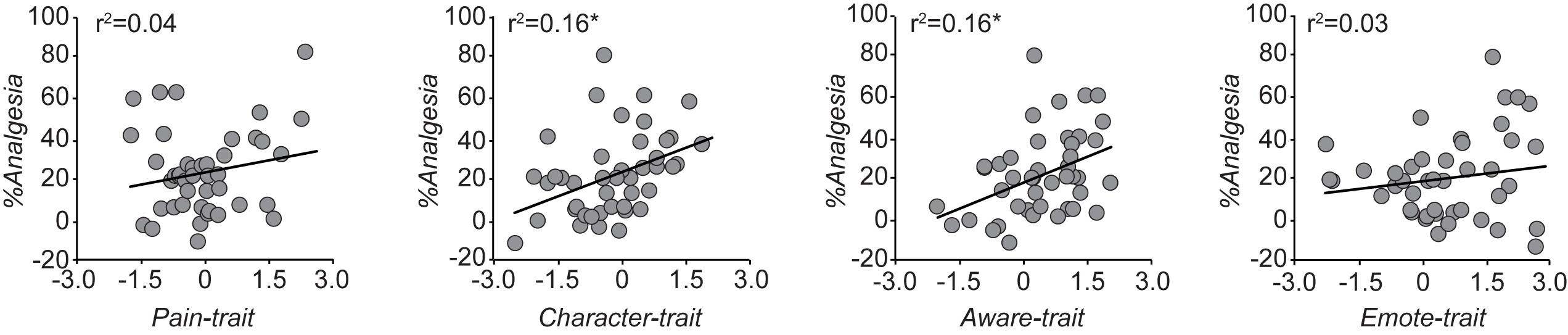

Supplement: S4 Fig — Character-trait and Aware-trait predisposed patients to the placebo response (% analgesia). Correlation P values were Bonferroni corrected for four comparisons *P < 0.05. (TIF) [file pbio.3000349.s004.tif]
