# Supplementary material for: Identification of traits and functional connectivity-based neurotraits of chronic pain
Source: PLoS Biol. 2019 Aug 20;17(8):e3000349. doi: 10.1371/journal.pbio.3000349 (PMC6701751; doi:10.1371/journal.pbio.3000349)
Supplement: S1 Table — Group 1 was on average 5 years younger than Group 2 (unpaired t test(106); t = 4.67, P = 0.03). There were no significant differences between the groups for gender, pain duration, or education level. (PDF) [file pbio.3000349.s005.pdf]

	Age (years)	Female (%)	Pain duration (months)	Education (years)
Group1	46.1 (12.5)	42%	55.0 (74.1)	13.7 (2.3)
Group2	50.8 (10.3)	43%	64.26 (67.94)	13.8 (2.1)
p_vals	0.03	0.87	0.51	0.74
