# Supplementary material for: Identification of traits and functional connectivity-based neurotraits of chronic pain
Source: PLoS Biol. 2019 Aug 20;17(8):e3000349. doi: 10.1371/journal.pbio.3000349 (PMC6701751; doi:10.1371/journal.pbio.3000349)
Supplement: S2 Table — Thirteen self-report measures (36 measures total if divided into respective subscales) were completed at designated visits in the study. The names and abbreviation are provided for each questionnaire, along with the rationale for why each measure was included in our battery. (DOCX) [file pbio.3000349.s006.docx]

| **Questionnaire** | **Visits Administered** | **Description** | **References** |
| --- | --- | --- | --- |
| CPAQ | 1 | *Chronic Pain Acceptance Questionnaire*: measures the effort participants put into either actively controlling their pain (activity engagement subscore) or passively accepting their pain (pain willingness subscore) | [1,2] |
| PCS | 1 | *Pain Catastrophizing Scale*: assesses how much people worry about their pain and its possible causes (yields a total score and subscales of rumination, magnification, and helplessness) | [3] |
| PASS-20 | all | *Pain Anxiety Symptoms Scale*: measures pain-related fear, avoidance, and anxiety | [4] |
| PSQ | 1 | *Pain Sensitivity Questionnaire:* assesses participants’ sensitivity to imagined painful and non-painful stimuli | [5] |
| MAIA | 1 | *Multidimensional Assessment of Interoceptive Awareness*: measures the extent to which someone is aware of his/her body and emotions and how well they can either focus or distract themselves from these sensations (8 subscales reflect various aspects of this awareness) | [6] |
| ERQ | 1 | *Emotional Regulation Questionnaire*: measures two kinds of strategies people use to control their positive and negative emotions (including a re-appraisal and a suppression subscore) | [7] |
| ACS | 1 | *Attentional Control Scale*: assesses the voluntary control of attention during a variety of situations | [8] |
| eACS | 1 | *Emotional Attentional Control Scale*: assesses the voluntary control of attention during emotionally demanding situations, which could include pain | [9] |
| FFMQ | 1 | *Five Facets of Mindfulness Questionnaire*: a combination of many well-known and validated questionnaires, this measures the five main components of mindfulness as a skill set. | [10] |
| NEO-FFI | 1 | *NEO Five* Factor Inventory: measures participants’ scores on personality dimensions (extraversion, agreeableness, conscientiousness, neuroticism, and openness); previous research has shown that neuroticism plays a role in chronic pain | [11,12] |
| LOT-R | 1 | *Life Orientation Test (Revised)*: measurement of dispositional optimism, which has been shown to influence placebo propensity in healthy individuals | [13] |
| LAQ | 1 | *Loss Aversion Questionnaire*: measures how sensitive participants are to a wide variety of potential “losses” in their lives. | [14] |
| PANAS | all | *Positive and Negative Affect Schedule:* assesses the extent to which participants are feeling a list of positive and negative emotions on the day of the visit to try to quantify the current affective state | [15] |

References

1. McCracken LM, Vowles KE, Eccleston C. Acceptance of chronic pain: component analysis and a revised assessment method. Pain. 2004;107: 159–66.

2. McCracken LM, Carson JW, Eccleston C, Keefe FJ. Acceptance and change in the context of chronic pain. Pain. 2004;109: 4–7. doi:10.1016/j.pain.2004.02.006

3. Sullivan MJL; B SR; Pivik, J. The Pain Catastrophizing Scale: Development and validation. Psychological Assessment. 1995;7: 524–532. doi:10.1037/1040-3590.7.4.524

4. McCracken LM, Dhingra L. A short version of the Pain Anxiety Symptoms Scale (PASS-20): preliminary development and validity. Pain research & management : the journal of the Canadian Pain Society = journal de la societe canadienne pour le traitement de la douleur. 2002;7: 45–50.

5. Ruscheweyh R, Verneuer B, Dany K, Marziniak M, Wolowski A, Colak-Ekici R, et al. Validation of the pain sensitivity questionnaire in chronic pain patients. Pain. 2012;153: 1210–8. doi:10.1016/j.pain.2012.02.025

6. Mehling WE, Price C, Daubenmier JJ, Acree M, Bartmess E, Stewart A. The Multidimensional Assessment of Interoceptive Awareness (MAIA). PloS one. 2012;7: e48230. doi:10.1371/journal.pone.0048230

7. Gross JJ, John OP. Individual differences in two emotion regulation processes: implications for affect, relationships, and well-being. Journal of personality and social psychology. 2003;85: 348–62.

8. Olafsson RP, Smari J, Guethmundsdottir F, Olafsdottir G, Harethardottir HL, Einarsson SM. Self reported attentional control with the Attentional Control Scale: factor structure and relationship with symptoms of anxiety and depression. Journal of anxiety disorders. 2011;25: 777–82. doi:10.1016/j.janxdis.2011.03.013

9. Barry TJ; H D; Lenaert, B; Debeer, E; Griffith, JW. The eACS: Attentional control in the presence of emotion. Personality and Individual Differences. 2013;55: 777–782.

10. Baer RA; S G T; Hoplins ,J; Krietemeyer, J; Toney, L. Using Self-Report Assessment to Explore Facets of Mindfulness. Assessment. 2006;13: 27–45.

11. Goldberg LR. The development of markers for the Big-Five factor structure. Psychological Assessment. 1992;4: 26–42.

12. Costa PT & McCrae, RR. Revised NEO Personality Inventory (NEO-PIR) and NEO Five Factor Inventory (NEO-FFI) professional manual. Odessa, FL; 1992.

13. Scheier MF; C Charles S,; Bridges, MW. Distinguishing optimism from neuroticism (and trait anxiety, self-mastery, and self-esteem): A reevaluation of the Life Orientation Test. Journal of Personality and Social Psychology. 1994;67: 1063–1078.

14. De Baets S& B M. Development of the loss aversion questionnaire [Internet]. 2012. Available: https://public.vlerick.com/Publications/3120e52a-f011-e211-96a6-005056a635ed.pdf

15. Watson D, Clark LA, Tellegen A. Development and validation of brief measures of positive and negative affect: the PANAS scales. Journal of personality and social psychology. 1988;54: 1063–70.
