# Supplementary material for: Identification of traits and functional connectivity-based neurotraits of chronic pain
Source: PLoS Biol. 2019 Aug 20;17(8):e3000349. doi: 10.1371/journal.pbio.3000349 (PMC6701751; doi:10.1371/journal.pbio.3000349)
Supplement: S3 Table — There were no differences in absolute scores of pain measurements between the groups, with the exception of physical health (SF-12p), which was higher in Group 1 than in Group 2. CBP, chronic back pain. (PDF) [file pbio.3000349.s007.pdf]

	Phone	Memory	NRS	MPQa	MPQs	Pain Detect	PANAS/n	BDI	SF-12p
Group 1	6.00 (1.21)	6.86 (1.49)	57.95 (22.16)	3.07 (2.87)	13.38 (5.66)	11.02 (6.20)	18.82 (6.47)	7.76 (5.94)	49.44 (9.25)
Group 2	n/a	n/a	50.56 (26.28)	3.28 (3.46)	12.52 (7.66)	11.00 (7.77)	16.57 (6.06)	7.62 (7.69)	40.23 (10.00)
p_vals	*n/a*	*n/a*	*0.12*	*0.72*	*0.50*	*0.99*	*0.07*	*0.92*	*<0.001*
