# Supplementary material for: Identification of traits and functional connectivity-based neurotraits of chronic pain
Source: PLoS Biol. 2019 Aug 20;17(8):e3000349. doi: 10.1371/journal.pbio.3000349 (PMC6701751; doi:10.1371/journal.pbio.3000349)
Supplement: S4 Table — (PDF) [file pbio.3000349.s008.pdf]

Figure panel	Tests
1b	Covariance matrix generated from Pearson correlations
1c	Network constructed from strongest 10% of correlations
1d	Principle Component Analysis were performed using varimax rotation making the components orthogonal
2a-b	Pearson correlations between each trait and pain measurements
3a	Variance explained using Pearson correlation between the chronic pain traits and the derived neurotraits in the test set
4a-b	Pearson correlations between chronic pain traits and neurotrait at each visit
5a	Principle Component Analysis were performed using varimax rotation making the components orthogonal
5b,c	Pearson correlations between each neurotrait and pain measurements
5d,e	Pearson partial correlations between Neurotrait 1 & 2 with and pain measurements after controlling for Neurotrait 3
5f,g	Pearson partial correlations between Neurotrait 3 with and pain measurements after controlling for Neurotrait 1 & 2.
6a	One-way ANCOVA for pain trait controlling for race/ethnicity and education: $F_{(3,44)} = 4.20$, $p = 0.01$
6b	Two sample ttest: $t_{(40)} = 2.30$; $p = 0.03$
6c	One-way ANOVA for pain trait controlling for ethnicity: *Neurotrait 1*, $F_{(3,40)} = 3.57$, $p = 0.02$; *Neurotrait 2,* $F_{(3,40)} = 2.62$, $p = 0.06$
6d	Spearman correlations between income and psychological factors
